# Subclinical hypocalcemia in dairy cows: reproductive and economic impacts on Eastern European farms

**DOI:** 10.3389/fvets.2025.1596239

**Published:** 2025-07-11

**Authors:** Daniel Ionut Berean, Liviu Marian Bogdan, Raluca Cimpean

**Affiliations:** ^1^Department of Reproduction, Faculty of Veterinary Medicine, University of Agricultural Sciences and Veterinary Medicine Cluj-Napoca, Cluj-Napoca, Romania; ^2^Department of Animal Breeding and Food Safety, Faculty of Veterinary Medicine, University of Agricultural Sciences and Veterinary Medicine Cluj-Napoca, Cluj-Napoca, Romania

**Keywords:** subclinical hypocalcemia, dairy cows, reproductive performance, mastitis, economic impact

## Abstract

**Introduction:**

This study investigated the association between postpartum subclinical hypocalcemia (SCHC) and reproductive and economic performance in dairy cows. SCHC is often overlooked yet may contribute significantly to reduced productivity and profitability in dairy herds.

**Methods:**

A total of 312 cows, Holstein Friesian and Romanian Spotted breeds, from three commercial farms in Eastern Europe were monitored during the first 60 days postpartum. Blood calcium levels were measured to classify cows into SCHC and normocalcemic groups.

**Results:**

The incidence of SCHC was 42.9%, with multiparous cows more frequently affected. Cows with SCHC had longer service periods, extended calving intervals, and required more artificial insemination attempts than normocalcemic cows. Although milk yield did not differ significantly between groups, SCHC cows exhibited moderately higher mastitis incidence and somatic cell counts, especially within the first 30 days postpartum. These factors contributed to increased indirect costs. Economic analysis indicated that SCHC cows incurred approximately 54 EUR more in additional costs per animal compared to normocalcemic cows.

**Discussion:**

SCHC is a hidden contributor to economic losses in dairy operations, primarily due to its negative impact on reproductive performance and udder health. These findings underscore the importance of early detection and preventive strategies to mitigate the effects of SCHC and improve herd productivity during the transition period.

## 1 Introduction

Calcium is a fundamental macromineral involved in maintaining the physiological homeostasis of dairy cows, playing key roles in neuromuscular function, hormone secretion, blood coagulation, immune responses, and bone metabolism (1). During the transition period, which spans from three weeks before to 3 weeks after calving, the calcium demand of dairy cows rises dramatically due to colostrum and milk production. This metabolic stress frequently leads to hypocalcemia, a disorder commonly divided into clinical (milk fever) and subclinical forms. While clinical hypocalcemia is easily recognizable through symptoms such as ataxia or recumbency, subclinical hypocalcemia (SCHC) presents no visible clinical signs and is often overlooked in farm settings (2, 3).

Despite its asymptomatic nature, SCHC has been associated with a range of significant reproductive and productivity issues. Studies have shown that up to 50% of multiparous dairy cows can experience SCHC postpartum, with a reduced prevalence in primiparous cows (4, 5). The pathophysiological mechanisms behind SCHC contribute to uterine atony, impaired immune function, delayed involution of the reproductive tract, and subsequent reproductive and infectious disorders. The condition has been consistently linked to the occurrence of retained placenta, endometritis, displaced abomasum, ketosis, and mastitis, among other disorders (4, 6, 7).

The reproductive impact of SCHC is well documented, as cows affected by calcium imbalances tend to exhibit longer service periods as well as prolonged calving intervals and decreased conception rates. These reproductive inefficiencies can significantly reduce a cow's lifetime productivity and profitability. Moreover, cows with SCHC are more prone to postpartum uterine diseases, which further compromise fertility and contribute to reproductive wastage (8, 9).

Another crucial aspect is the role of SCHC in predisposing cows to mastitis, one of the most economically significant diseases in the dairy industry. The immune dysfunction associated with SCHC facilitates bacterial colonization of the mammary gland, leading to increased rates of clinical and subclinical mastitis, as well as elevated somatic cell counts (SCC) in milk an indicator closely monitored in dairy quality control systems. High SCC values not only reduce milk quality but are also associated with penalties from milk processors and regulatory bodies (10, 11).

From an economic perspective, the burden of SCHC extends beyond veterinary treatment costs to encompass decreased milk production, lower reproductive efficiency, higher culling rates, and the loss of genetic potential through early removal of valuable cows from the herd. Although these associations have been extensively studied in North America and parts of Western Europe, research in Eastern and Central Europe remains sparse. Dairy systems in these regions often present distinct management practices, nutritional regimens, and housing systems that may influence both the prevalence of SCHC and its consequences (12, 13).

To address this knowledge gap, the current study was conducted in three commercial dairy farms located in Eastern Europe. The farms involved manage Holstein-Friesian and Romanian Spotted Breed cows under intensive production systems. The study aimed to investigate the correlations between postpartum SCHC and reproductive performance parameters (service period, calving interval, conception rate), mastitis occurrence, somatic cell counts (SCC), and the associated economic impact.

## 2 Materials and methods

### 2.1 Animals

The study was conducted on three commercial dairy farms: one located in southern Hungary (Hódmezovásárhely region) and two in Satu Mare County, western Romania. The farms were selected based on similarities in herd size, housing conditions, and general management practices, all operating under intensive dairy production systems. While general management systems were comparable, differences existed in specific practices such as milking systems and somatic cell count detection methods.

A total of 312 postpartum dairy cows were enrolled in the study, including Holstein-Friesian and Romanian Spotted breeds. Of the 312 cows, 128 originated from the Hungarian farm, while 184 were distributed between the two Romanian farms. Both multiparous and primiparous cows were included to represent the diversity typically found in commercial herds. The animals were housed under standard commercial conditions common to the region, primarily in free-stall barns with access to outdoor exercise areas when available.

Feeding management consisted of a total mixed ration (TMR) formulated to meet the nutritional requirements of dairy cows in early lactation. The TMR comprised approximately 60% forage dry matter, including maize silage (40%), grass silage (15%), and hay (5%), and 40% concentrate dry matter, consisting of grains, protein sources, and mineral-vitamin premixes. Mineral premixes, including calcium, phosphorus, and vitamin D3, were added to the concentrate (2%) to ensure appropriate levels of essential nutrients supporting calcium metabolism. Water was available ad libitum throughout the study. The health status of all animals was monitored routinely by farm veterinarians, and only clinically healthy cows without evident metabolic or infectious diseases were enrolled in the study to reduce confounding effects on calcium metabolism.

### 2.2 Calcium determinations

Blood samples were collected from each cow at three time points postpartum: within the first hour after calving, at 48 h postpartum and on day 10 postpartum.

Samples were obtained via coccygeal venipuncture using lithium-heparinized vacutainers to avoid calcium-binding anticoagulants such as EDTA. Ionized calcium (iCa) concentrations were measured directly on-farm using the LAQUAtwin CA-11C calcium ion meter (Horiba, Japan), which allowed rapid determination of iCa values in the field.

For diagnostic purposes, cows with iCa concentrations below 2 mmol/L were classified as having subclinical hypocalcemia (SCHC) (12). Animals with iCa values above this threshold were categorized as normocalcemic. Measurements were repeated at each time point to ensure accuracy, and calibration of the device was performed every 10 samples according to manufacturer recommendations.

### 2.3 Reproductive indicators

Throughout the postpartum monitoring period, reproductive performance data were collected for all enrolled cows. The study focused on key parameters such as the service period and the calving interval. Additionally, conception rates were recorded, taking into account cows that achieved pregnancy during the follow-up period. Another important metric included the average number of artificial inseminations required to establish pregnancy, reflecting the reproductive efficiency of each animal. All reproductive information was retrieved from the computerized management systems implemented on the participating farms. Only cows that completed the entire follow-up period and remained in production without being culled for others reproductive or health reasons were included in this study.

### 2.4 Mastitis and somatic cell count (SCC) determination

Udder health monitoring was carried out through the identification of clinical mastitis cases and the systematic evaluation of subclinical mastitis using somatic cell count (SCC) determinations. Clinical mastitis diagnoses were based on veterinary examinations, focusing on local inflammatory signs in the udder and changes in milk quality.

For the detection of subclinical mastitis, SCC measurements were performed at pre-established postpartum intervals across all three farms. On the Hungarian farm, SCC values were automatically obtained via sensors integrated into the robotic milking system. On the two Romanian farms, SCC analyses were performed using dedicated SCC analyzers installed in the milking parlors, which allowed real-time assessment of milk samples during routine milking.

SCC determinations were performed at 7, 15, 30, and 60 days postpartum for each enrolled cow, regardless of breed or parity. Subclinical mastitis was diagnosed when SCC values exceeded 200,000 cells/mL. The SCC results were subsequently used to analyze correlations with subclinical hypocalcemia status and reproductive performance recorded in the study.

A threshold of 200,000 cells/mL was set to define subclinical mastitis, following international guidelines (14). Cows exceeding this threshold were classified as subclinical mastitis cases. The collected SCC data were subsequently integrated with other health and production parameters for further analysis.

### 2.5 Economic calculations

These calculations included costs related to veterinary treatments, reduced milk yield, extended service periods and calving intervals, as well as potential culling and replacement expenses for non-recovering animals. The economic evaluation was designed to capture both direct and indirect costs. Direct costs included therapies for mastitis, endometritis, or retained placenta, reproductive inefficiencies reflected in increased artificial insemination expenses due to prolonged time to conception, as well as culling and replacement costs for cows culled due to chronic health problems associated with SCHC (15). Indirect costs encompassed milk production losses and penalties for elevated somatic cell counts (16, 17). The specific values and rates applied in the economic assessment are detailed in Table 1.

**Table 1:** Estimated costs associated with SCHC.

**Parameter**	**Unit cost**	**Notes**
Artificial insemination	20.00 EUR/attempt	Increased costs for cows with prolonged service periods or low conception rates
Milk yield loss	1.20 EUR/cow/day	Loss of income due to decreased milk production during delayed reproductive recovery
Veterinary treatment (Mastitis)	35.00 EUR/case	Includes antibiotics, anti-inflammatories, and labor for clinical mastitis treatment
Culling and replacement cost	1,200.00 EUR/animal	Cost for herd replacement following premature culling due to chronic mastitis or infertility
SCC milk quality penalty	0.02 EUR/liter	Applied when SCC exceeds 200,000 cells/mL, resulting in milk processor penalties

### 2.6 Statistical analysis

The data collected during the study were organized and processed using Microsoft Excel for preliminary analysis and IBM SPSS Statistics (Version 26.0) for advanced statistical evaluation. The normality of the datasets was assessed using the Shapiro-Wilk test to determine the appropriate statistical methods for comparison. For variables with a normal distribution, parametric tests were applied to compare means, while non-parametric tests were used for data that did not meet the normality criteria. Relationships between categorical variables, such as the occurrence of mastitis and the presence of subclinical hypocalcemia, were examined using Chi-square tests. The analysis also focused on identifying significant differences in reproductive performance indicators, such as service period length and number of inseminations, as well as milk production parameters and somatic cell counts, between cows diagnosed with subclinical hypocalcemia and normocalcemic cows. The effects of breed and farm location on the prevalence of hypocalcemia and related disorders were also explored. Statistical significance was set at a *p* < 0.05 throughout the analysis.

## 3 Results

### 3.1 Incidence of SCHC

**Figure 1:**
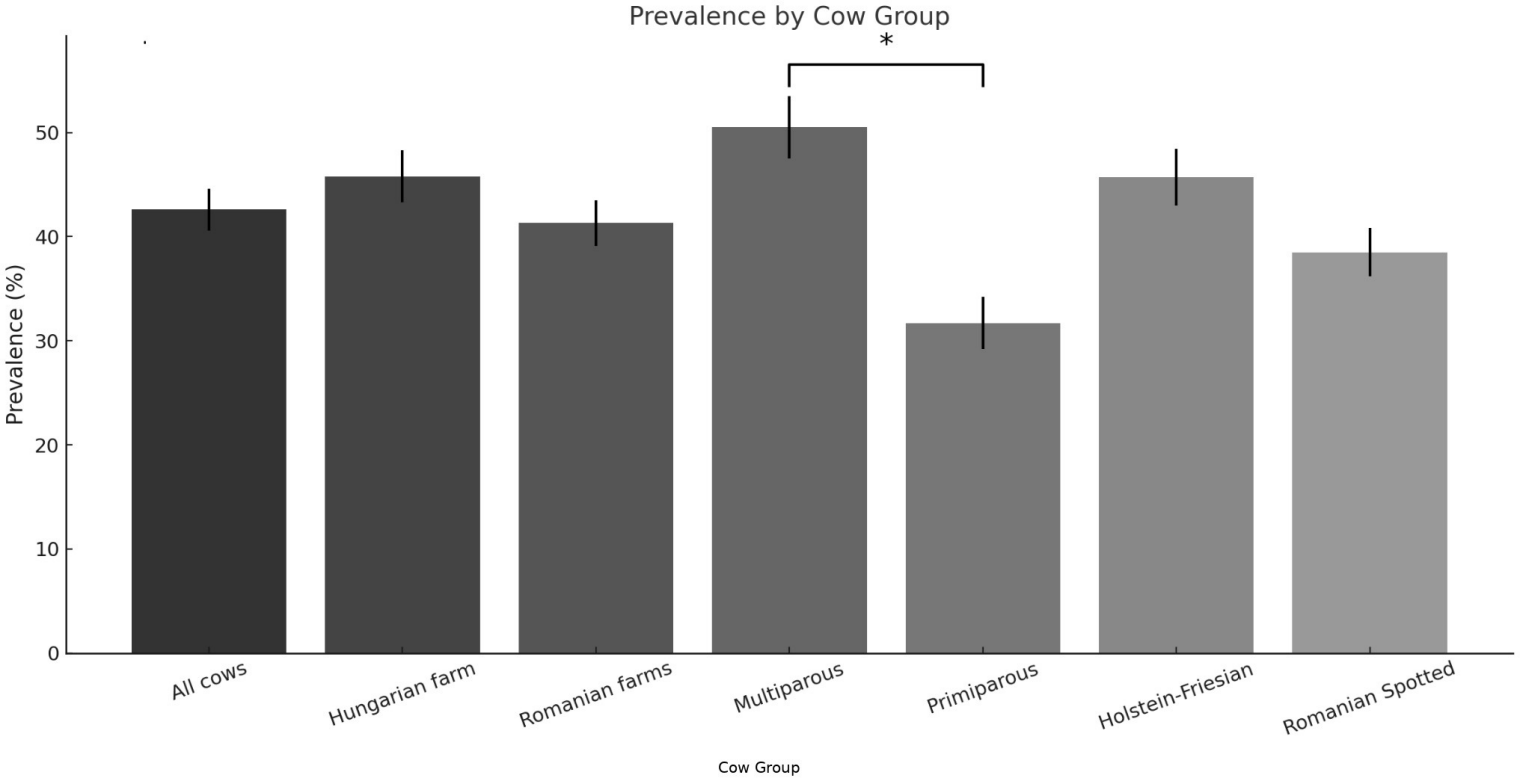
Prevalence of subclinical hypocalcemia by category. ^*^Multiparous cows had the highest SCHC prevalence, which was significantly higher than that of primiparous cows, indicating that parity is a key risk factor. Other categories showed statistically non-significant differences.

From the total of 312 cows enrolled in the study, 134 cows (42.9%) were diagnosed with subclinical hypocalcemia during the postpartum period, based on calcium determinations. Among these, 94 cases (70.1%) occurred in multiparous cows, while 40 cases (29.9%) were identified in primiparous cows. Breaking down the SCHC incidence by location, the Hungarian farm (*n* = 128) recorded 58 SCHC cases, representing 45.3% of its sampled population. In the two Romanian farms combined (*n* = 184), 76 cows were affected, equating to a 41.3% prevalence rate. Regarding breed differences, out of 190 Holstein-Friesian cows monitored, 87 (45.7%) developed SCHC, whereas in the Romanian Spotted breed group (*n* = 122), 47 cows (38.5%) were affected. The prevalence of subclinical hypocalcemia was significantly higher in multiparous cows compared to primiparous cows (*p* < 0.01), confirming parity as a risk factor. However, no statistically significant differences were observed between the Hungarian and Romanian farms (*p* = 0.48) or between the two breeds studied (*p* = 0.15) (Figure 1).

### 3.2 Reproductive indicators

The analysis revealed that cows diagnosed with subclinical hypocalcemia had a statistically significant extension of the service period and calving interval compared to normocalcemic cows (*p* < 0.05). Additionally, the SCHC group required more artificial insemination attempts to achieve pregnancy. However, daily milk yield did not differ significantly between groups (*p* = 0.31), suggesting that metabolic status had a more pronounced effect on reproductive efficiency than on overall production (Table 2).

**Table 2:** Reproductive performance in SCHC vs. normocalcemic cows.

**Reproductive parameter**	**SCHC group (mean ±SD)**	**Normocalcemic group (mean ±SD)**	***p*-value**	**Statistical significance**
Service period (days)	92.0 ± 13.5	78.0 ± 11.2	< 0.05	Significantly longer in SCHC group
Calving interval (days)	412.0 ± 18.6	397.0 ± 15.9	< 0.05	Significantly longer in SCHC group
Artificial inseminations per conception	1.8 ± 0.5	1.4 ± 0.4	< 0.05	Significantly higher in SCHC group
Daily milk yield (kg)	33.7 ± 3.2	34.1 ± 3.0	0.31	No significant difference

### 3.3 Mastitis and somatic cell count

Cows affected by subclinical hypocalcemia presented a higher occurrence of both clinical and subclinical mastitis compared to normocalcemic animals. Among the SCHC group, 36 cases of clinical mastitis were recorded (26.8%), while in the normocalcemic group, 31 cases (17.4%) were identified.

Subclinical mastitis, based on somatic cell count (SCC) measurements, also showed a tendency toward higher values in the SCHC group. At 30 days postpartum, the average SCC in SCHC cows reached 248,000 cells/mL, compared to 210,000 cells/mL in normocalcemic cows. By 30 days postpartum, SCC values decreased in both groups, with SCHC cows averaging 205,000 cells/mL and normocalcemic cows averaging 183,000 cells/mL (Table 3).

**Table 3:** Mastitis incidence and somatic cell count results.

**Parameter**	**SCHC group (mean ±SD)**	**Normocalcemic group (mean ±SD)**	***p*-value**	**Statistical significance**
Clinical mastitis cases (%)	36/134 (26.8%)	31/178 (17.4%)	0.06	Tendency toward higher incidence in SCHC group
Peak SCC (cells/mL at 30 days)	248,000 ± 52,000	210,000 ± 45,000	0.04	Mildly significantly higher SCC in SCHC group
SCC at 60 days postpartum (cells/mL)	205,000 ± 49,000	183,000 ± 41,000	0.09	No significant difference

### 3.4 Economic values

The economic impact analysis demonstrated that cows affected by subclinical hypocalcemia generated higher cumulative costs over the six-month postpartum period compared to normocalcemic cows, largely due to additional reproductive interventions and health management.

In the SCHC group, the increased number of artificial inseminations combined with prolonged service periods led to additional insemination-related expenses, amounting to an average of 8 EUR more per cow compared to normocalcemic animals. Veterinary treatment costs were also moderately higher in the SCHC group, as these cows required more frequent interventions for mastitis and reproductive disorders. On average, SCHC cows incurred 18 EUR more in veterinary costs than normocalcemic cows.

Milk yield differences contributed a small but notable component to the economic disparity. Despite similar daily milk yields between groups, SCHC cows tended to experience more frequent transient production drops due to health events such as mastitis, resulting in an estimated additional loss of approximately 9 EUR per cow over the study period.

Culling and replacement costs also showed a difference, with a slightly higher culling rate in SCHC cows due to reproductive or chronic udder health issues. The additional financial impact from culling was calculated at an average of 15 EUR per SCHC cow when distributed across the entire group.

Altogether, the cumulative additional cost per cow associated with SCHC was estimated at ~50 EUR over the six-month period when compared to normocalcemic cows (Table 4).

**Table 4:** Economic comparison between SCHC and normocalcemic cows.

**Cost parameter**	**SCHC group (EUR/cow)**	**Normocalcemic group (EUR/cow)**	**Difference (EUR)**
Artificial insemination costs	36.0	28.0	+8.0
Veterinary treatment costs	53.0	35.0	+18.0
Milk yield loss (due to health disorders)	11.0	2.0	+9.0
Culling and replacement cost (averaged)	21.0	6.0	+15.0
SCC milk quality penalty	6.0	2.0	+4.0
**Total additional cost per cow**	**127.0**	**73.0**	**+54.0 EUR**

The bold values represent the total additional costs per cow for each group, highlighting the overall economic impact of health conditions in the SCHC group compared to the Normocalcemic group.

## 4 Discussion

Subclinical hypocalcemia (SCHC) remains a significant concern in modern dairy production systems due to its subtle but multifaceted impact on animal health, reproductive performance, and farm economics. Although not clinically apparent like milk fever, SCHC has been recognized as a risk factor for several postpartum disorders and is increasingly linked to production inefficiencies. The present study aimed to evaluate the incidence of SCHC and its correlations with reproductive indicators, mastitis occurrence, somatic cell counts, and economic outcomes in dairy cows managed under Eastern European commercial farm conditions.

The incidence of SCHC found in this study (42.9%) aligns with global estimates, which suggest a prevalence between 30% and 50% in multiparous dairy cows during early lactation (18, 19). The slightly higher occurrence in multiparous cows compared to primiparous cows (50.5% vs. 31.7%, p < 0.01) is consistent with reports indicating that older cows have reduced bone calcium mobilization efficiency and diminished hormonal responses to calcium demand (2, 12, 20). The lack of significant differences between farm locations or breeds suggests that SCHC prevalence may be more influenced by physiological factors such as parity and transitional metabolic demands than by external farm conditions (21).

The study revealed that SCHC cows experienced longer service periods and calving intervals compared to normocalcemic cows (*p* < 0.05), in line with research by Martinez et al. (22), who found that calcium imbalances impair uterine contractility and delay uterine involution, predisposing cows to postpartum uterine infections and prolonged anestrus periods. Moreover, the higher number of artificial inseminations required in the SCHC group (1.8 vs. 1.4, *p* < 0.05) is consistent with findings from Caixeta et al. (9), who associated SCHC with reduced conception rates and suboptimal reproductive outcomes. The reproductive inefficiencies observed in SCHC cows are likely multifactorial, resulting from both direct effects on smooth muscle function and indirect effects through increased susceptibility to uterine infections (23).Interestingly, while reproductive delays were statistically significant, the differences were moderate, suggesting that the farms involved in this study may have employed effective postpartum management strategies to minimize fertility losses.

The higher incidence of clinical mastitis (26.8% in SCHC vs. 17.4% in normocalcemic cows, p = 0.06) and the elevated SCC levels (p = 0.04 at 30 days postpartum) in SCHC cows observed in this study mirror results reported by Rodriguez et al. (24) and Ghasemi et al. (25). These studies linked subclinical hypocalcemia with immune suppression and reduced neutrophil functionality, which increase susceptibility to intramammary infections. However, by 60 days postpartum, SCC levels between groups did not differ significantly (*p* = 0.09), suggesting partial recovery of mammary gland health as the lactation progressed. This pattern aligns with Várhidi et al. (26), who observed that SCC peaks are often transient in early lactation SCHC cows when appropriate mastitis control practices are implemented. The moderate nature of these differences, particularly the borderline *p*-value for clinical mastitis incidence (*p* = 0.06), may reflect farm-specific factors such as good milking hygiene, routine SCC monitoring, and early mastitis intervention protocols, which likely mitigated the impact of SCHC on udder health.

The economic analysis underscored the hidden costs of SCHC, with affected cows generating approximately 54 EUR more in expenses per animal than normocalcemic cows during the six-month period. This aligns with estimates from McArt et al. (27), who highlighted how SCHC increases herd-level costs due to fertility delays, veterinary interventions, and culling risks. The greatest contributors to this economic burden were reproductive costs (insemination and extended service period) and health-related expenses, particularly treatments for mastitis and reproductive disorders. Although milk yield differences were not statistically significant between groups (*p* = 0.31), the indirect effects of health disorders, notably mastitis, contributed to milk quality penalties, reinforcing findings by Esnaola et al. (28), who emphasized the importance of considering milk quality deductions in the economic evaluation of metabolic disorders.

While SCHC does not dramatically alter milk output in the short term, it compromises reproductive efficiency and udder health, leading to cumulative financial losses. The findings also emphasize the need for effective preventive strategies, such as optimizing the dietary cation-anion difference prepartum and early calcium supplementation postpartum (29, 30). These results contribute to the growing body of evidence supporting the economic importance of metabolic health monitoring in transition cows, especially in large commercial dairy farms operating under intensive production models.

## 5 Conclusion

SCHC is a common metabolic disorder in dairy cows during the postpartum period, particularly affecting multiparous animals across both Holstein-Friesian and Romanian Spotted breeds. Although no significant differences in SCHC prevalence were observed between the studied farms in Hungary and Romania, its impact on reproductive performance, udder health, and economic outcomes was evident. Cows affected by SCHC showed extended service periods, prolonged calving intervals, and required more insemination attempts to achieve pregnancy compared to normocalcemic cows. Additionally, a moderate increase in both clinical and subclinical mastitis incidence was observed in the SCHC group, contributing to higher somatic cell counts and subsequent milk quality penalties. While daily milk yield was not significantly altered, the indirect effects of SCHC on fertility and udder health led to cumulative financial losses over the postpartum period.

From an economic standpoint, SCHC cows generated approximately 54 EUR more in costs per animal over 6 months, driven primarily by reproductive inefficiencies, veterinary treatments, and increased culling rates. Overall, this study highlights the importance of early detection and proactive management of SCHC in dairy herds, particularly in multiparous cows. The findings support the integration of preventive nutritional strategies and routine metabolic monitoring to reduce the hidden costs of this condition and improve herd productivity and profitability.

## Data Availability

The raw data supporting the conclusions of this article will be made available by the authors, without undue reservation.
